# The Nature and Function of Digital Information Compression Mechanisms in the Brain and in Digital Television Technology

**DOI:** 10.3389/fnsys.2016.00040

**Published:** 2016-05-06

**Authors:** John Smythies, Maximilien d'Oreye de Lantremange

**Affiliations:** ^1^Laboratory of Integrative Neuroscience, Department of Psychology, Center for Brain and Cognition, University of CaliforniaSan Diego, La Jolla, CA, USA; ^2^Schorey BVBABrussels, Belgium; ^3^Resultance SALuxembourg, Luxembourg

**Keywords:** vision, parallel processing, digital television, information compression technology, cortical injuries, virtual reality, object perception

## Why compare human vision with digital television?

Both of these systems perform similar functions. In both, light rays carrying information are reflected off objects and are picked up by a camera-like device and transmitted to a complex mechanism that performs computations on them. In television these computations construct a representation of the objects in the form of the pictures on the TV screen. This representation needs to be optimized for the viewer (i.e., the viewer should be pleased with the resolution and frame rate and ideally should perceive the same as when viewing natural objects). In vision the computations construct the phenomenal objects that experienced in a person's visual field in consciousness—as Crick (1994, p. 159) described it “We have for example a vivid internal picture of the external world.” The brain actually has multiple “TV screens,” i.e., multiple visual maps, which “look” at each other. So, here again, there is sort of TV screen, which should be optimized for the viewer. At the end reciprocal communications give rise to consciousness.

Recently a key information compression mechanism has been discovered in the brain that operates in a manner very similar to the same mechanism used in digital television (Nortmann et al., 2013): for details of this see below. So a comparative study of both systems may have three useful consequences.

- It may reveal to what extent skillful TV engineers and Darwinian evolution come up with similar solutions to the basic problem of making the visual representational mechanisms more efficient.
- Discoveries of how the natural system works may give clues to digital TV designers for new mechanisms to try in their system.
- Likewise neuroscientists might look for any evidence that further mechanisms used by TV engineers also occur in the brain.

## Brain mechanisms

We will first review what is known about the mechanisms whereby the brain processes visual information starting at the macroanatomical level. The visual system in the cortex is organized into two streams—the dorsal and the ventral (Ettlinger, 1990; Haxby et al., 1991; Gooddale and Milner, 1996). The dorsal goes from V1 to the parietal lobe and deals with visually guided behavior (“where” and “how”). The ventral goes from V1 to the temporal lobe and deals with object recognition and identity (“what”). These authors called these the “perception” and “action” pathways. The subtle relations between the concept of “where” and how mechanisms on the one hand and the “perception” and “action” pathways on the other have been addressed by Lebedev and Wise (2002). Then there is the color pathway that goes from V1 to V4 in the temporal lobe, and the motion pathway that goes from V1 to V5 in the middle temporal lobe.

It has long been established that the brain's visual system processes information from the retina by three anatomically and functionally separate parallel distributed pathways that mediate color, form (Gooddale and Milner's ventral stream) and motion. Gooddale and Milner's dorsal path deals with organizing visually guided behavior and not perception. This tripartite system is supported by the evidence on how vision returns after injuries to the visual cortex. Schilder (1942) reports that the first faculty to return is pure motion, usually rotary, without any color or form. Then “space” or “film” colors appear floating in space. This is followed by objects, often only in parts such as the handle of a teacup. Lastly complete objects are seen into which the colors enter.

In order to avoid unnecessary complications that tend to result from trying to translate directly between the technical terminology used in neuroscience and that used in digital television, we propose to use a neutral terminology as follows. The tripartite systems used in both the consciousness-related system in the brain and in digital TV may be identified as composed of the color system (V4), the motion system (V5), and a system that deals the construction of visual phenomenal objects (Object Construction System or OCS). These three systems may function independently, as in the cases reported by Schilder (1942). They can also be selectively deleted leaving only the other two to function. For example, in neuroscience, the color system is deleted in achromatopsia where the subject sees everything in black and white (Sacks, 1997). Thus, the OCS system can be identified as a black and white processing system. Cases in which motion is selectively deleted, or illusionarily added, have been reported in experiments using hallucinogens such as mescaline (Smythies, 1953). In one such case the subject reported,

“The perception of a moving burning cigarette was a great surprise to me. Not a continuous line or circle was seen, as under normal conditions in the dark room, but a number of small glowing balls. At the end of the movement I could see the entire movement as it were fixed by a number of glowing balls standing in the air. Then these balls jumped all of a sudden in a great hurry into the glowing end of the cigarette. They did not fade, but all of them went along the curve to the terminal point just as if they were connected by a rubber band…

…I saw that the scales of the fish as well as the fish itself were distinctly moving. [They were at dinner.] I was unable to eat it. I admired the certainty with which Dr. B. was convinced of the death of the fish. The noodles behaved literally and without exaggeration as a moving heap of worms.”

Objects can be deleted, as in stage 2 of Schilder's data in which objects are not seen but only “space” colors and motion. We recognize that the “object construction system” (OCS) may differ in details between the brain and digital TV.

Mainly on the basis of a series of psychophysical experiments, that paired color with motion or color with orientation, Zeki (2015) has proposed that the brain is a massively asynchronous organ and has no central (master) clock that resets the activity in each of its parallel systems. Thus, in the visual system color, OC and motion are processed independently resulting in an asynchronous behavioral output from each independently.

Evidence that the brain actually contains one of these information compression mechanisms will be detailed below.

## Information compression mechanisms used in digital television

It is therefore not surprising that modern digital television has developed tripartite signal compression technologies that appear to use much or some of, the same system as the brain. In summary, a video compression system first separates information relating to black and white images from color information. The former provides the main source of information needed to construct the structure of the visual images on the TV screen as an OCS. It is possible to code adequate color information using much fewer “bits” than black and white information. This explains why black and white and color are processed separately in the system. Color processing in the brain is faster than form and motion processing because there are fewer bits to transmit. The visual compression system used here is the MPEG 2 standard (ISO/CEI 13818-2 and 3). Many other compression methods and standards use similar techniques. MPEG 2 uses YUV encoding (Y being luminance and UV the 2 chroma components). It is preferred over RGB precisely because it allows compressing chrominance more efficiently (with less bits). For background information on TV compression technology see Beach (2010).

It might be argued that in the emerging era of very high broadband transmissions, the encoding schemes such as MPEG (and other more recent types) are quickly becoming obsolete as the required compression is no longer so critical as a few years ago

However, this argument is invalid for two reasons:

- The amount of video being produced (smartphones, web cams, video surveillance, etc.) is increasing exponentially. This video material needs to be stored and/or transmitted over networks like the Internet. Without compression the storage would be impossible or prohibitively expensive and the networks would be completely saturated. Computation engineers have invented video compression to cope with vast and increasing amounts of information (and it is only getting bigger and bigger), so it is likely that the brain has done the same thing.
- The resolution of the video material is getting higher and higher. Television networks first went from standard SDTV (780 × 420 pixels) to high definition HDTV (1920 × 1080), and are now talking about 4K (4096 × 2060). The new Iphone 6 can already shoot videos in 4K, and some broadcasters are envisioning Ultra High Digital TV (UHDTV), which is 7680 × 4320 pixels. A single UHDTV picture contains the equivalent of 100 SDTV pictures. Take that in 3D and the amount of information would be doubled.

To compress the signal even further, the system removes temporal redundancy. In other words, it removes information that is the same from one picture to the next. The most common way of doing this is motion estimation. Instead of sending all the pictures with all the details, the system transmits only some pictures with all the details, and then only transmits information about changes in the other images. This is a completely different process that the two previous ones, because it is not based on transformation in the frequency domain. Instead, the image is divided in small elements and the system computes in which directions these elements are individually moving from one picture to the next. This process is much more complex and computation intensive than the two previous ones. The resulting information is coded into “motion vectors” and transmitted to the receiving device). In the receiving device, the system needs to put the 3 types of information together in order to reconstruct the video image.

It is of great interest therefore that experimental evidence has been reported that suggests that such a mechanism exists in the visual brain. Nortmann et al. (2013) conducted voltage-sensitive dye imaging experiments in the visual cortex. The presentation consisted of vertically and horizontally filtered natural images. The authors found that at 33 Hz the encoding represented the current input. *In contrast at low frequencies (10 Hz) the encoding represented, not the currently exposed images, but the difference in orientation between consecutive images*. That is to say if these individual images were presented at 33 Hz (30 ms per image), the neurons represented complete image information. But at 10 Hz (100 ms), the neurons represented only those elements that were new or missing, that is, image differences. Thus, the authors concluded that at slow frequencies (10 Hz) V1 no longer reports actual features but reports instead on their relative distance over time. The authors state,

“When compared with the preceding image, the cortical activity patterns characterized exactly the difference in orientations. Consequently, large amounts of incoming data were relatively suppressed, reminiscent of differencing methods (Fowler et al., 1995) used for video data compression in communication technology.” The authors go on to suggest that possible functions of this system relate to error detection, or comparison of current output with predictions of what that output should be based on previous performance (see also Carlson et al., 2016). The latter process is well-researched under the name of “predictive coding” (Huang and Rao, 2011).

These authors say,

“Predictive coding is a unifying framework for understanding redundancy reduction and efficient coding in the nervous system. By transmitting only the unpredicted portions of an incoming sensory signal, predictive coding allows the nervous system to reduce redundancy and make full use of the limited dynamic range of neurons.”

We would stress here the information compression aspect of prediction coding that has been somewhat neglected. It also seems possible that this form of information compression may be used in other brain areas than the visual system (Table 1).

**Table 1:** **Tabulation of main similarities and differences between digital TV mechanisms and brain visual mechanisms**.

	**Digital television**	**Brain mechanisms**
Virtual reality	Yes	Yes
Data compresion	Yes	Yes
Three channels	Yes	Yes
Lateral inhibition	No	Yes
Modal plasticity	No	Yes
Motion vectors	Yes	Unknown

*Lateral inhibition refers to the process by which visual neurons inhibit neighboring neurons to increase sharpness of vision. Modal plasticity refers to the fact the modal function of a neuron can be changed in natural circumstances, e.g., from being a visual neuron to being a somatosensory neuron, by epigenetic mechanisms involving its afferent neuronal input*.

In correlating color, OC and motion the TV system does not have the problem of differential speed that the brain has. Today's processors are fast enough to do all the necessary computation (black and white, color and motion) related to a single frame (even at very high resolution) in the time period between two consecutive frames. However, because motion compensation also requires information from previous and following frames, the system needs to buffer a number of frames before being able to process a given frame. The buffering takes place at the compressions side as well as the decompression side. For that reason a video transmission system that includes motion compensation always has a delay of a few frames.

In the brain, signal compression is partly effected by mixing direct “reality” signals to the visual cortex from the retina with “virtual reality” signals from cortical memory banks that supply a proportion of the invariant images between successive perceptions. This includes the filling-in of scotomata (Ramachandran and Gregory, 1991), during saccades (Kleiser et al., 2004), and retinal rivalry experiments in binocular vision (Kovács et al., 1996; see Smythies, 2009 for details).

MPEG 2 video compression relies on information stored in memory. For example, during shooting footage and while the camera stays still, one only needs to transmit once the information that remains static in the picture. This is called an Intra frame (I frame). This information is stored at the receiving side. For the following pictures, the system will only send information about moving parts of the picture. The decoder will use this information in combination with stored information in order to reconstruct the full picture. This type of picture is called a Predicted (P) frame. In MPEG 2 one can even do this using parts of past pictures as well as future pictures. It is called Bi-directional (B) frames.

Other video compression standards go even further and consider that an image is a collection of objects. Each object can be compressed and encoded with its own technique. MPEG 4 already relies on this idea. Ultimately a decoder could rely on its own database of objects instead of the transmission of object descriptions. The database could contain static objects, objects stored for a long time, or objects that are received just before they are used. The system would then only need to send descriptions of new objects and motion information, the decoder would reconstruct the picture using its database. This type of technology requires large amounts of processing power and memory capacity, so tradeoffs need to be made in function of what is physically available and what processing delay is acceptable. These tradeoffs often result in quality limitations. From what the experiments demonstrate, both video compression and the brain seem to do this kind of tradeoff.

Video systems do more than compare small aligned portions of subsequent video frames. They also take small parts of the reference frame and search in a much large area in the predicted frame if the same information is also present. If the same information is found in the predicted frame, this information is not transmitted again, but instead only the relative position of the information in the new frame compared to where it was in the reference frame is transmitted (this is called motion estimation, and the information transmitted is called a motion vector). It is not known if the brain possesses any mechanism similar to this, although this would be worth looking for.

This “object focused” technique has an interesting cross-link with a recent development in neuroscience. Jankowski and O‘Mara (2015) have reported the existence in the claustrum and adjacent limbic cortex of “object” cells. These respond to a particular object in the environment (e.g., a glass vase or a bunch of flowers) irrespective of its shape or color. This also links with Pylyshyn's Visual Index Theory (Pylyshyn, 2001). He suggests that the brain does not first ascertain the properties of objects and then coordinates these properties into objects. Instead, he proposes, that the brain first allocates attention to objects *qua* objects and only later takes cogniscence of their properties. This process involves the primary detection and tracking of objects and thus functions as an OCS.

## Conclusion

This paper has made the case that there may be at least some similarities between brain mechanisms, and mechanisms used in information compression technologies in TV, relating to the existence and nature of the three information-processing pathways used in each. Important experimental evidence to support the existence of one type of information compression mechanism in the brain has been detailed.

We suggest therefore that there are three main similarities between the brain's mechanisms and those used by digital TV

- use of virtual reality mechanisms.
- use of coding by removal of temporal redundancy.
- use of three different computational channels for color, form and motion.

There are of course many differences between the two systems including those in the nature of the electrical codes being used and the physical structure of the actual computing mechanisms. Further progress in this direction needs experiments to test the hypothesis. We have commenced a search of other systems in the brain to see if there is evidence that they also employ the temporal redundancy displacement system used in the visual system.

The parallels between the brain and digital video systems may open new perspectives of research in both fields. Digital video systems may give new hints on how the brain uses motion estimation in order to reduce the amount of information to be processed. The better understanding of foveated vision and the brain mechanisms that process information about objects could help engineers design even more efficient compression algorithms.

Note that image compression in television based on foveated vision has already been studied (Wang and Bovik, 2005) but not, as far as we know, put into commercial use.

## Author contributions

JS contributed ideas and the neuroscience part. MD contributed ideas and the television technology part.

### Conflict of interest statement

The authors declare that the research was conducted in the absence of any commercial or financial relationships that could be construed as a potential conflict of interest.
